# Quercetin: a natural compound for ovarian cancer treatment

**DOI:** 10.1186/s13048-019-0530-4

**Published:** 2019-06-15

**Authors:** Rana Shafabakhsh, Zatollah Asemi

**Affiliations:** Research Center for Biochemistry and Nutrition in Metabolic Diseases, Kashan University of Medical Sciences, Kashan, IR Iran

**Keywords:** Ovarian cancer, Quercetin, Genetic alterations

## Abstract

Ovarian cancer is the main cause of death among all reproductive cancers in females. In 2018, ovarian cancer was the seventh most common cancer of women entire the world. A wide variety of molecular and genetic alterations as well as different response to therapies in the different types of ovarian cancer lead to problems in design a common therapeutic strategy. Besides, ovarian cancer cells have tendency to acquire resistance to common cancer treatments through multiple mechanisms. Various factors, including cytokines, growth factors, proteases, adhesion molecules, coagulation factors, hormones and apoptotic agents have been examined to find effective cancer treatment. Phytochemicals have been indicated to have great potential anti-cancer properties against various types of cancers. Quercetin is one of the phytochemicals that exists extensively in daily foods. Wide evidences revealed that quercetin is able to inhibit various types of cancers including breast, lung, nasopharyngeal, kidney, colorectal, prostate, pancreatic, and ovarian cancer. Several in vitro and in vivo studied conducted to evaluate cytotoxic effects of quercetin on ovarian cancer. Since quercetin does not harm healthy cells and it is cytotoxic to cancer cells via various mechanisms, researchers suggest that it could be an ideal agent for ovarian cancer treatment or an adjuvant agent in combination with other anti-cancer drugs. Thus, in this review, we focused on chemo-preventive and curative attitude of quercetin for ovarian cancer and summarize some of the most recent findings which regard the possible molecular mechanisms by which this natural compound inhibits this cancer.

## Introduction

Ovarian cancer is the most fetal of all reproductive cancers, the eleventh most common type, and the fifth main cause of cancer-associated death in women. In 2018, ovarian cancer was the seventh most frequent cancer of females globally, with about 240.000 new subjects [1]. Ovarian cancer is commonly not diagnosed until advanced stages because of its silent and obscure symptoms which make it hard to cure appropriately [2]. In spite of widespread awareness of this cancer in these years, its survival rate has not significantly changed due to difficulties existing in its early diagnosis [3]. Some common symptoms of ovarian cancer are including abdominal pain, abdominal bloating, urinary frequency and changes in bowel habits [2, 4]. It is essential for health care supporters to consider these vague and non-specific symptoms especially in high-risk cases. Several risk factors, including family history or genetic predisposition, ovulation, endometriosis, dietary factors, and race has been known for this disorder [5]. Ovarian cancer divided into 3 types: epithelial (most frequent), germ cell, and sex-cord-srtomal. Epithelial ovarian cancer has four histological subtypes: serous, endometrioid, mucinous and clear cell [2]. The various molecular and genetic alterations of these types of ovarian cancer as well as their different responses to therapies lead to a challenge in design a common treatment strategy [6]. In ovarian cancer, the tumor microenvironment is consist of immune cells, fibroblasts, extracellular matrix (ECM), some enzymes such as matrix metalloproteinase (MMPs), and growth factors such as vascular endothelial growth factor (VEGF), transforming growth factor- β (TGF-β), and platelet-derived growth factor (PDGF). These components promote tumor cell proliferation, migration and invasion [7].

Ovarian cancer cells are willing to establish resistance to common cancer therapies. Cancer cells are able to acquire drug-resistance via multiple mechanisms [8]. A large number of factors, including inflammatory cytokines, growth factors, proteases, adhesion molecules, coagulation factors, hormones, and apoptotic agents have been evaluated in order to find effective cancer treatment. Wide experimental studies have demonstrated that phytochemicals such as polyphenols, flavones and flavonoids exert great potential anti-cancer properties against various types of cancers [9].

Quercetin is one of the phytochemicals that is widely found in foods consumed daily. This polyphenol compound widely exists in nuts, teas, vegetables, herbs and generally daily diet of people [10]. Also, it is available as commercial supplement. It is safe at oral dosages of 1 g/day which is absorbed up to 60% [11]. Quercetin has an extended variety of pharmacological usages such as antioxidant, anti-diabetic, anti-inflammatory and anti-proliferative functions [12–14]. Quercetin, 2-(3,4-dihydroxyphenyl)-3,5,7-trihydroxy4H-chromen-4-one, is well known by its 2-hydroxyflavone backbone consisted of two benzene rings, A and B, linked by a 3-carbone heterocyclic pyrone one [13]. The strong ability of quercetin in free radical scavenging and binding to transition metal ions is due to the presence of two antioxidant pharmacophores in its structure [13]. In addition, the presence of catechol and the OH group at position C3 of its structure provide a great configuration for scavenging of free radicals [13, 15]. Quercetin is a pentalhydroxyflavonol which has 5 hydroxyl groups on its flavonol skeleton at 3, 30, 40 5, and 7 position carbons. Various biochemical and pharmacological functions of quercetin result from the substitution of its different functional groups [16]. Several investigations showed that quercetin could be present in two ways: free state or aglycone and conjugated by various molecules such as carbohydrates, lipids, alcohols, and sulfate group which produce quercetin derivatives, including quercetin glycoside, prenylated quercetin, quercetin ethers and quercetin sulfate [16]. Moreover, a large amount of research on this bioactive compound has proposed several pathways which interact to treat cancer [17, 18]. Several evidences revealed the inhibitory effects of quercetin against wide variety types of cancers, including breast, lung, nasopharyngeal, kidney, colorectal, prostate, pancreatic, and ovarian cancer [19–22]. Previous studies have reported that consumption of quercetin-high rich vegetables was related to a decreased risk of ovarian cancer [23–25]. Likewise, consumption of fruits contains quercetin such as apples and citrus fruit juices reduce the incidence of ovarian cancer [26]. Several studies have investigated cytotoxic effects of quercetin on ovarian cancer cells both in vitro and in vivo. While quercetin do not harm healthy cells, it has been identified to be cytotoxic to cancer cells via various mechanisms suggesting to be an ideal agent for ovarian cancer treatment or an adjuvant agent in combination with other anti-cancer drugs [27]. Thus, in this review, we focused on chemo-preventive and curative attitude of quercetin for ovarian cancer and summarize some of the most recent findings which regard the possible molecular mechanisms by which this natural compound inhibits this cancer.

### Ovarian cancer carcinogenesis and progression: molecular mechanisms

There are several theories about the processes by which ovarian cancer progresses. One of them is ovulation theory suggests that frequent ovulation with recurrent breakdown and repair of ovarian epithelium elevates the possibility of DNA damage and carcinogenesis [28]. Therefore, more ovulations provide higher risk of ovarian cancer development. Hyper-ovulation in rats significantly elevates the possibility of ovarian adenocarcinoma progression. Some experimental studies revealed that ovulation may lead to carsinogenesis by initiating multiple cellular events [29]. Hence, excessive ovulation increases the risk of mutagenicity which may be intervened by inflammation. Various inflammatory mediators such as prostaglandins and leukoteriens as well as vasoactive substances such as bradykinin are increased during ovulation. Follicle rupture which occurs during ovulation has also features of inflammation. Taken together, the process of ovulation is strongly associated with inflammatory reactions. Especially, epithelium located around the ovulation site, have more contacts with inflammatory mediators such as cytokines, prostaglandins and oxidative stress [30]. Another theory is gonadotrophin theory which suggests that increased levels of gonadotrophins induce epithelium proliferation within inclusion cysts directly or by the stimulation of steroidogenesis leading to neoplastic transformation [31]. Data also reported that reproductive hormones are related to ovarian cancer carcinogenesis as progestins which have pro-apoptotic actions, reduce the risk of tumor transformation while estrogen promotes malignancy [32]. Moreover, chronic inflammation has also been suggested to play potential role in ovarian cancer development [33]. Epithelial ovarian cancer cells are able to metastasize to the peritoneal cavity and lead to an accumulated ascites which provide an immunosuppressive microenvironment exacerbating tumor development. Ascetic fluid activates protein kinase B (or Akt) and prevents apoptosis induced by tumor necrosis factor receptor apoptosis-inducing ligand (TRAIL) [34]. The dynamic alteration in cytokines of ascites has been indicated in a recent study in which ascites and plasma samples of advanced epithelial ovarian cancer showed a remarkable change in cytokines and inflammatory molecules [35]. Moreover, a wide variety of inflammatory agents, including interleukin-6 (IL-6), Akt, lysophosphatidyl acid (LPA) and protein kinase C (PKC) have been found to be elevated in ovarian cancer [36]. LPA is identified to elevate IL-6, IL-8 and VEGF through Akt/ nuclear factor kappa B (NF-kB) pathway in ovarian cancer cells [37]. PKC also plays a vital modulatory role in a large number of signal transduction pathways of cancer. Impairment in PKC function has been observed in tumorogenesis and drug-resistance related to ovarian cancer [38]. Altogether, these mentioned agents and pathways actively enhance ovarian cancer related to inflammation. Inflammation naturally generates several toxic oxidant agents which causes directly damage of DNA, proteins, and lipids leading to carcinogenesis [39]. Additionally, chronic inflammation is related to elevated cell proliferation. Excessive and rapid cell division causes additional replication errors resulting in DNA repair and thereby may elevate mutagenesis risk [40]. Ovarian cancer cells release several inflammatory mediators, cytokines and interleukins [41]. For example, prostaglandins levels are greater in ovarian tumors than normal cells [42]. Elevated levels of prostaglandins stimulate the invasion of cancer cells [43]. Another important event which is developed during ovarian cancer is oxidative stress. Findings have reported that patients with ovarian cancer have reduced concentrations of antioxidants and greater levels of oxidative stress than healthy women [44–47]. In previous studies, it has been observed that epithelial tissues of ovarian cancer exert a pro-oxidant condition with elevated expression of pro-oxidant enzymes and reduced expression of antioxidant enzymes. Elevated expression of inducible nitric oxide synthase (iNOS), myeloperoxidase (MPO), NAD(P) H oxidase, and nitric oxide (NO) as well as lowered apoptosis have been observed in ovarian cancer tissues [45, 48, 49]. Moreover, increased nitrosylation of caspase-3 which leads to a significant reduction in the activity of caspase-3 was seen in ovarian tumors. MPO is an important oxidant enzyme which enhances the production of NO from iNOS [50, 51]. Many recent data reported an elevation in MPO levels of ovarian cancer cells [52]. MPO also has key roles in the regulation of apoptosis, immune surveillance mechanisms, 3-nitrotyrosine formation, and inflammatory responses [45]. Additionally, MPO as a source of free iron promotes oxidative stress through contributing to reactive oxygen species (ROS) generation. Under oxidative stress, iron reacts with hydrogen peroxide (H2O2) and thereby increases ROS production [47]. Above all, oxidative stress may play a key role in ovarian cancer maintenance and progression.

### Anti-cancer effects of quercetin

Quercetin is a lipophilic compound which is able to cross the cellular membranes and initiate multiple intracellular signaling pathways implicated in chemoprevention. Several investigations have reported the potential anti-cancer effects of quercetin in wide variety types of cancers. One of the unique abilities of quercetin is its dual function as a pro-oxidant or antioxidant [53]. A ROS elevation result from oxidative stress leads to DNA damage which is promotes mutations. Mutations lead to uncontrolled growth of malignant tumor cells. Quercetin can reduce ROS by donating electrons and thereby decreased ROS-mediated DNA damage [10, 54]. This is the primary antioxidant mechanism of quercetin observed at its cellular concentrations which could be obtained by diet [55]. In contrast, quercetin increases oxidative stress and cytotoxicity at higher concentrations by elevating damage and inducing apoptotic pathways in tumor cells [10, 54]. Quercetin is also known as a great apoptosis inducer at its high concentrations. It is proved that mitochondrial-mediated pathway is the main mechanism which quercetin used for its apoptosis induction effect [56–58]. Quercetin induces apoptosis through activation of p53, elevation of pro-apoptotic molecules such as Bax, caspase-3, caspase-9 and reduction of anti-apoptotic agents such as survivin and Bcl-2 [59]. Several experimental studies revealed that higher dosages of quercetin are also able to induce apoptosis via death-domain pathways in various cancer cells [60]. In addition, emerging data is showing that quercetin directly inhibits protein chaperons and thereby stimulates apoptosis [61, 62]. Protein chaperons have role in protein folding and maintenance that disruption in their performance leads to cell death. Un-regulation of heat shock protein (HSP) chaperones is induced by ionizing radiation in many tumor cells. Recent findings revealed that quercetin inactivates protein chaperons by inhibiting the kinases which contribute to HSP induction [62]. Thus, quercetin may be applied as an adjuvant in combination with current chemotherapies in cancer treatment. Quercetin is also observed to arrest cell cycle in several types of cancers. Quercetin down-regulates cyclin D1/Cdk4 and E/Cdk2 and up-regulates p21 and thereby induces the G1 cell cycle arrest [63]. Similarly, in vitro studies have reported that quercetin induces cell cycle arrest in G2/M, G0/G1 and G2/M phases [20, 21]. Moreover, quercetin arrests cell cycle in the S phase by inhibiting DNA synthesis [64]. Quercetin-induced up-regulation of p21, p27, p53 and Chk2 along with down-regulation of Cdk1 and cyclin B1 leading to cell cycle arrest in the G1 and G2/m phase, have been observed in various cancer cell lines [65, 66].

Quercetin is able to directly bind tubulin by which induce depolymerization of cellular microtubules leading to cell cycle arrest [67]. Thus, quercetin affects a wide variety of molecules involved in the cell cycle process. PI3K-Akt/PKB pathway which is involved in several processes such as cell survival regulation, cell cycle and growth progression, and also carcinogenesis [68], can be targeted by quercetin in various types of cancers resulting in apoptosis induction and inhibition of cancer initiation and development [69]. Furthermore, tyrosine kinases are a kind of cancer-related molecules which play role in the transduction of growth factors and oncogenes. Recent findings revealed that quercetin inhibits tyrosine kinases leading to inhibition of the growth of tumor cells [70, 71]. Besides, p53 is a key molecule in the anti-cancer and pro-apoptotic effects of quercetin. Several studies have reported that quercetin induced phosphorylation and stabilizing of p53 levels. For instance, in HepG2 cells quercetin contributed to p53 expression and phosphorylation leading to cell cycle arrest and apoptosis induction [72]. Moreover, in colon carcinoma cells, quercetin and p53 contribution also resulted in increased expression of NAG-1 and thereby increased apoptosis [73]. Also, P53 increases p21 levels attenuating the pro-apoptotic effects of quercetin in cancer cells. On the other hand, p53 has antioxidant function by regulating of several genes, including microsomal GSH transferase homolog PIG12, Gpx1, aldehyde dehydrogenase ALDH4A1, Mn-SOD2, and catalase [74–76]. In oxidative stress condition, p53 is essential for decreasing intracellular ROS concentrations. Additionally, decreased p53 sensitizes cells to damage of H2O2 which leads to decreased viability, increased apoptosis and elevated DNA oxidation. However, in cancer cells p53 functions are significantly blocked by down-regulating of its gene. So quercetin may improve ROS elevation induced by p53 deficiency through elevating of its levels [77]. Taken together, these findings highlight the significant effects of quercetin in cancer treatment. Apoptosis induction, pro-oxidant and antioxidant actions, cell cycle arrest induction, regulation of several cancers related proteins such as p53, HSPs, and tyrosine kinases are some mechanisms recently reported by which quercetin can inhibit cancers.

### Quercetin and ovarian cancer

Recently, the effects of quercetin on ovarian cancer have been interesting for many researchers. In this way, several experimental studies have been conducted to elucidate the mechanisms by which quercetin inhibits ovarian cancer. Liu Y et al. [78] investigated the effect of quercetin on apoptosis in mice with ovarian cancer. The results showed that quercetin induced apoptosis via the mitochondria intrinsic and caspase-dependent pathways. Besides, quercetin evoked endoplasmic reticulum (ER) stress in ovarian cancer resulting in mitochondria-mediated apoptosis. Furthermore, quercetin was able to induce autophagy which has a protective role in ovarian cancer cells. Taken together, this study indicated that quercetin induced ER stress, apoptosis and authophagy via a p-STAT3/Bcl-2 axis. Another investigation reported that quercetin decreases the viability and induces apoptosis of metastatic ovarian cancer cells. Quercetin decreases several anti-apoptotic molecules, including Bcl-2 and Bcl-xL and increases pro-apoptotic molecules, including caspase-3, caspase-9, Bid, Bax, Bad and cytochrome c. In addition, quercetin inhibits the growth of metastatic ovarian cancer cells by the induction of mitochondrial-mediated apoptosis [79]. In a recent study, anti-cancer effects of nano-formulation of quercetin were examined. This form of quercetin significantly inhibited growth of ovarian cancer cells both in vitro and in xenograft ovarian cancer mice. Furthermore, nano-formulated quercetin induced apoptosis by activating caspase-3, caspase-9 and Bax as well as reducing MCL-1 and Bcl-2 [80].

Several experiments have examined the synergistic effect of quercetin in combination with other chemotherapeutic agents against ovarian cancer. Gong c et al. [81] assessed the effect of quercetin in combination with irradiation on ovarian cancer in an in vitro*/*in vivo study. Quercetin treatment led to ER stress, increased expression of p53, p21 and Bax, decreased expression of Bcl-2, prolonged DNA repair as well as induced radio-sensitization in ovarian cancer cells. In human ovarian cancer xenograft model, quercetin in combination with radiation significantly inhibited tumor growth and activated p53. In another recent report, quercetin pretreatment strongly potentiated cisplatin cytotoxicity in ovarian cancer. Quercetin increased ER stress, inhibited STAT3 phophphorylation, downregulated Bcl-2 expression in ovarian cancer cells. In xenograft mous model of ovarian cancer, quercetin also increased anti-tumor effects of cisplatin. In mice treated with cisplatin in combination with pretreatment of quercetin, lowered Bcl-2 and higher apoptosis were observed compared with other groups. This study suggested that quercetin may be a good candidate for adjuvant therapy in ovarian cancer treatment [82]. Another form of quercetin, PEGylated liposomal quercetin (Lipo-quercetin) was evaluated on cicplatin-sensitive and cisplatin-resistant human ovarian cancer models both in vitro and in vivo. In vitro investigations reported that Lipo-quercetin suppressed cell growth and induced apoptosis and cell cycle arrest in both tumor cell types. Experiments in ovarian cancer xenograft models of mice showed that Lipo-quercetin more inhibited tumor proliferation compared with free quercetin. Moreover, Lipo-quercetin induced apoptosis in in vivo study [83]. Likewise, another in vitro study supported the synergistic effect of quercetin in combination with cisplatin in ovarian cancer cells [84]. Some studies have investigated the influences of quercetin on cell cycle progression. The cell cycle modulation and cytotoxic effect of quercetin on ovarian cancer cells was investigated in an in vitro study. Findings showed that quercetin decreased the expression of cycline D1 without influence on cyclin B1 which could be associated with G1/S phase alteration [85]. In an experimental study, quercetin could inhibit proliferation and induce apoptosis in ovarian cancer cells in a dose manner. The protein levels of survivin were decreased when the quercetin dose increased. Quercetin also led to cell cycle arrest in G0/G1 phase and G2/M phase [86]. Another study revealed that quercetin may arrest the cell cycle via inhibiting 1-phosphatidylinositol 4-kinase (PI kinase) activity and reducing inositol-1,4,5-triphosphate (IP3) levels [87]. Tumor necrosis factor-related apoptosis-inducing ligand (TRAIL) has been proven to have antitumor activity in several cancer types including ovarian cancer. Yi L et al. [88] evaluated the effect of quercetin on sensitization of ovarian cancer cells to TRAIL. Results indicated that quercetin elevated the sensitization of tumor cells to TRAIL leading to reduced expression of cell survival proteins, inhibited tumor growth and increased pro-apoptotic proteins including caspase-3. Scambia et al. [89] demonstrated that quercetin inhibited growth of ovarian cancer cells via modulating transforming growth factor beta 1 (TGF-beta-1) production. Results indicate that quercetin increased TGF-beta-1 activity. Northern blot analysis revealed no alteration in TGF-beta-1 mRNA levels suggesting that TGF-beta-1 modulation occurs at posttranscriptional levels. In an in vitro study, the effect of methylquercetin, 3,4′,7-O-trimethylquercetin (34′7TMQ) on proliferation, invasion and migration of ovarian cancer cells were measured. In mentioned study, the expression of proliferating cell nuclear antigen (PCNA) urokinase plasminogen activator (uPA), plasminogen activator inhibitor-1(PAI-1) and MMP-2 were analyzed. The results indicated that 34′7TMQ inhibited the migration and the invasion of ovarian cancer cells but had no effect on cell proliferation. After 34′7TMQ treatment, the expression of uPA and MMP-2 also inhibited, but PAI-1 and PCNA levels were not changed [90]. In contrast to above results which revealed the beneficial effects of quercetin in ovarian cancer treatment especially in combination with other treatments, a recent experimental study reported that quercetin lowered the effects of some anti-neoplastic drugs including cisplatin, 5-fluorouracil, taxol, and pirarubicin. Low concentrations of quercetin had anti-apoptotic effect and decreased ROS-mediated injury of anti-neoplastic drugs and also increased antioxidant enzymes in tumor cells [91]. Taken together, these findings have demonstrated that quercetin exerts antitumor features against ovarian cancer via several mechanisms (Table 1).

**Table 1:** Experimental studies that investigated the role of quercetin in ovarian cancer

Form of quercetin	Doses	Type of cervical cancer	Model	Findings	Ref
Quercerin	80 mg/kg twice a week	–	In vivo	Induced apoptosis, induced ER stress, activated p-STAT3/ Bcl2 axis, induced protective authophagy,	[78]
34′7TMQ	–	Epithelial and fibroblast ovarian cancer cell lines	In vitro	Inhibited cell migration and invasion, inhibited expression of uPA and MMP-2	[90]
Quercetin	A dose range	Metastatic ovarian cancer cell line	In vitro	Decreased viability, induced apoptosis, decreased Bcl-2 and Bcl-xL, increased caspase-3, caspase-p, Bid, Bad, Bax, cytochrome c	[79]
Quercetin	100 μM	Multi-drug resistant ovarian cancer cell line	In vitro	Increased ER stress, prolonged DNA repair, increased expression of p53, p21 and Bax, decreased expression of Bcl-2, induced radio-sensitization,	[81]
Quercetin	–	Human ovarian cancer xenograft model	In vivo	increased radiation-induced cell death, increased p53, increased ER stress	
Quercetin	A dose range	Cisplatin sensitive and resistant cell lines	In vitro	Enhanced cisplatin cytotoxicity, increased ER stress, suppressed STAT-3 phosphorylation, decreased expression of Bcl-2,	[82]
	40 mg/kg once a week	Human ovarian cancer xenograft model	In vivo	Suppressed STAY-3 phosphorylation, decreased Bcl-2 expression, induced apoptosis	
Quercetin	A dose range	Epithelial ovarian cancer cell line and its CIS-resistant cell line	In vitro	Decreased expression of cyclin D1	[85]
Quercetin	A dose range	Epithelial ovarian cancer cell line	In vitro	Inhibited proliferation, induced apoptosis, decreased survivin, induced cell cycle arrest	[86]
Nano-formulation of quercetin	A dose range	Ovarian endometrioid adenocarcinoma	In vitro	Inhibited growth, induced apoptosis, activated caspase-3 and caspase-9, decreased expression of MCL-1 and Bcl-2, increased expression of Bax	[80]
	A dose range	Human ovarian cancer xenograft model	In vivo	Inhibited growth, induced apoptosis, inhibited angiogenesis	
Quercetin	A dose range	Epithelial and drug resistant ovarian cancer cell lines	In vitro	Decreased ROS, increased anti-oxidant enzymes, inhibited apoptosis	[91]
		Human ovarian cancer xenograft model	In vivo	Increased anti-oxidant enzymes expression, reduced ROS, decreased anti-neoplastic drug’s efficacy	
Lipo-Que	–	CIS-resistant and CIS-sensitive ovarian cancer cell lines	In vitro	Inhibited proliferation, induced apoptosis, induced cell cycle arrest	[83]
		Human ovarian cancer xenograft model	In vivo	Inhibited tumor growth, inhibited proliferation, induced apoptosis	
Quercetin	2 mg / kg	Human ovarian cancer xenograft model	In vivo	Increased TRAIL sensitization, inhibited tumor growth, increased caspase-3, induced apoptosis	[88]
Quercetin	10 microM	Ovarian serous adenocarcinoma	In vitro	Increased TGF beta 1 activity	[89]
Quercetin	A dose range	Metastatic ovarian serous adenocarcinoma	In vitro	Inhibited PI kinase, decreased IP3 levels	[87]

## Conclusions

**Fig. 1:**
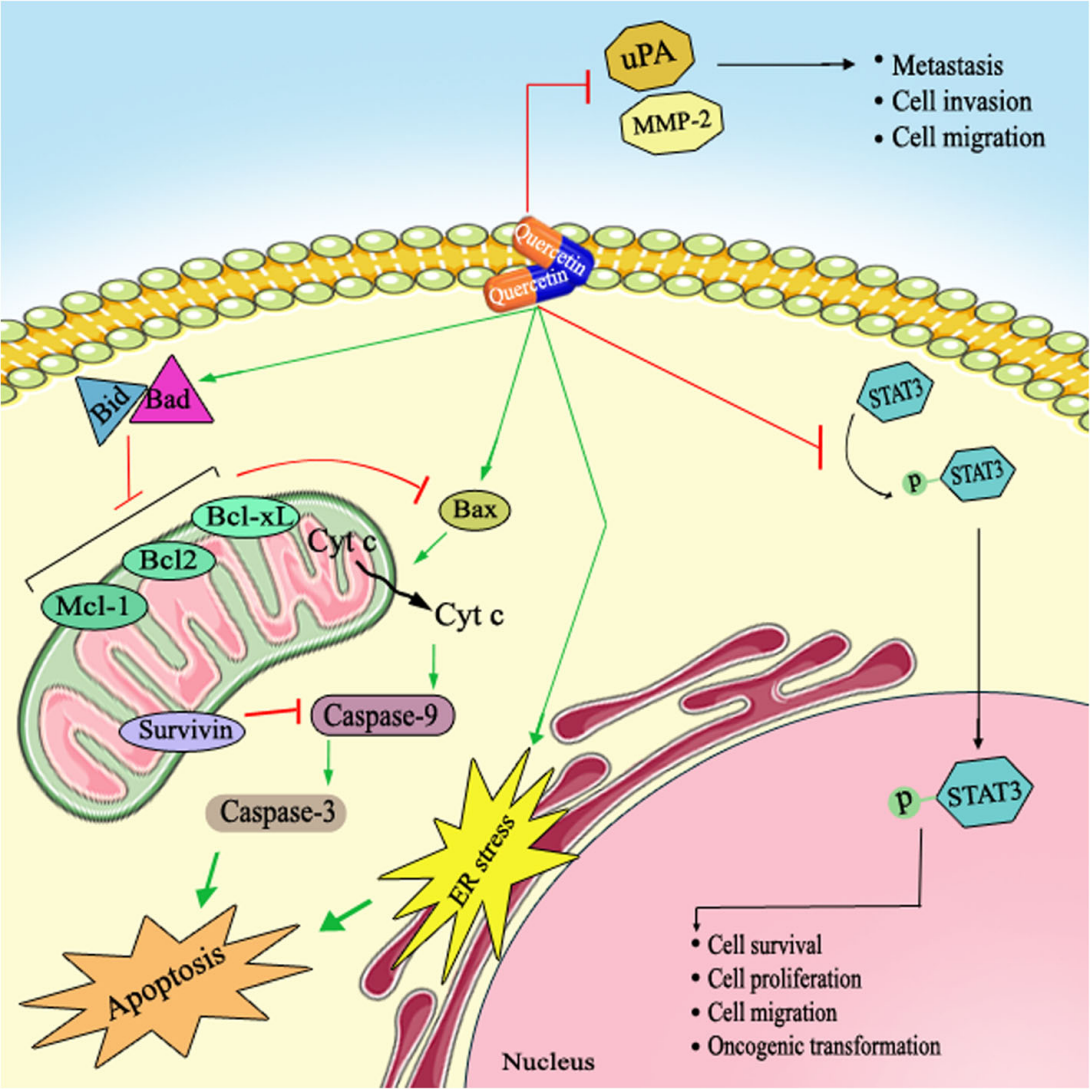
Schematic representation in targeting different signaling pathways using quercetin as a novel therapeutic strategy in the treatment of ovarian cancer

In etiology of ovarian cancer, several molecular mechanisms, including inflammation, oxidative stress and DNA damage are complicated. Findings reported that quercetin inhibits ovarian cancer by its anti-inflammatory, pro-oxidative, anti-proliferation, apoptosis induction, and cell cycle arrest induction activities. Besides, this natural compound can intensify the effects of other chemo-therapeutic drugs (Table 1 and Fig. 1). However, more investigations are required to completely elucidate its exact mechanisms of action against ovarian cancer. Collectively, quercetin may be a good candidate for ovarian cancer treatment especially in combination with other chemo-preventive drugs due to its potential synergistic effects.

## Data Availability

The primary data for this study is available from the authors on direct request.

## Abbreviations

ECM: extracellular matrix
MMPs: matrix metalloproteinase
PDGF: platelet-derived growth factor
TGF-β: transforming growth factor- β
VEGF: vascular endothelial growth factor
